# Disparities in Employer-Provided Benefits Among Older Black and Hispanic Workers

**DOI:** 10.1093/geroni/igaf122.445

**Published:** 2025-12-31

**Authors:** Bruna Lopez, Cal Halvorsen

**Affiliations:** Boston College, Chestnut Hill, Massachusetts, United States; Washington University in St. Louis, St. Louis, Missouri, United States

## Abstract

Employer-provided benefits, while important for workers across the life course, may be particularly essential to older workers due to the higher cost of private health insurance for older adults, increased attention to retirement savings, and need for flexibility for caregiving responsibilities. In response, we analyzed nearly 30,000 observations of workers between the ages of 50 and 64 using six waves of the biennial Health and Retirement Study (HRS), from 2010 to 2020 and HRS sample weights to promote the representativeness of our results. We documented trends in access to employer-provided health insurance, retirement savings programs, and the ability to reduce working hours overall and by gender, race, and ethnicity. We found that about 60% of workers had access to employer-provided health insurance, about 60% of workers had access to employer-sponsored retirement savings programs, and about 30% of workers reported the ability to reduce their work hours. Rates were relatively consistent throughout the study period. Although male and White workers are more likely to have access to these benefits than female and Black workers, as well as those in the “all other races” category, the largest differences were by ethnicity: Hispanic older workers reported access to employer-sponsored retirement savings programs, for example, at rates up to 30 percentage points less than their non-Hispanic counterparts. This disparity may be attributed to workplace culture differences. Overall, results suggest that Hispanic older workers are the least likely to have access to employer-provided benefits, potentially foreshadowing more health and financial difficulties later in life.

